# Event-Driven Spiking Neural Networks for Private Vehicle Parking Prediction

**DOI:** 10.3390/e28030253

**Published:** 2026-02-25

**Authors:** Wangchen Long, Jie Chen

**Affiliations:** 1 College of Artificial Intelligence, Zhuhai City Polytechnic, Zhuhai 519090, China; longwangchen@zhcpt.edu.cn; 2 School of Advanced Interdisciplinary Studies, Hunan University of Technology and Business, Changsha 410205, China; 3 Xiangjiang Laboratory, Changsha 410205, China

**Keywords:** mobility prediction, spiking neural networks, event-driven modeling, irregular temporal sampling

## Abstract

Predicting the future parking locations and durations of private vehicles using vehicular edge devices is critical for real-time intelligent transportation services, ranging from instant point-of-interest recommendations to dynamic route planning. Advanced deep neural networks like Transformers demonstrate exceptional performance in mobility prediction; however, their heavy reliance on dense matrix multiplication makes them unsuitable for real-time applications on vehicular edge devices. Spiking neural networks offer a potential solution due to their asynchronous event-driven characteristics and low power consumption. However, existing spiking neural networks face three fundamental challenges: (1) handling heterogeneous inter-event intervals; (2) mitigating quantization errors in regression tasks under limited simulation steps; and (3) efficiently regulating information flow based on external contexts. To address these challenges, we propose an event-driven spiking neural network for private vehicle parking prediction called Spark. First, we design a Time-Adaptive Leaky Integrate-and-Fire neuron with a lookup table-based decay mechanism to efficiently model variable inter-event intervals. Second, an accumulate-based readout strategy is introduced to mitigate quantization errors by integrating discrete spike trains into continuous output values for high-precision regression. Third, a Spiking Contextual Gating module is proposed to selectively regulate spiking information flow across channels based on environmental context. These components are integrated into a unified architecture that maintains high prediction accuracy while remaining computationally efficient. Extensive experiments on real-world datasets demonstrate that Spark achieves an effective balance between prediction accuracy and computational efficiency compared to baselines.

## 1. Introduction

Private vehicle parking prediction aims to model and forecast stationary event sequences such as parking location and duration of stay based on the historical vehicle trajectories and contextual information. Accurate private vehicle parking prediction will benefit a wide spectrum of applications, including personalized point-of-interest (POI) recommendation services [1], dynamic route planning [2], and refined electric vehicle charging scheduling strategies [3]. For example, by predicting the driver’s intended destination and parking duration, vehicle edge systems such as smart cockpits can pre-load navigation routes or suggest relevant services based on a short drop-off or long-term parking, thereby ensuring a seamless and intelligent user experience.

Due to the high degree of freedom and variation of human movement [4], it is difficult to predict future parking locations and durations for private vehicles. Early works leveraged sequential statistical models (e.g., Markov chains) [5] to capture transition probabilities for mobility prediction but failed to model high-order dependencies. Recent research has made significant progress in leveraging deep neural networks to capture sequence patterns, such as Recurrent Neural Networks (RNNs) [6,7] and Transformers [8]. However, the deployment of these advanced models faces a hardware dilemma in the real world. Transformers achieve high accuracy but impose heavy computational loads. Their quadratic complexity causes unacceptable latency on commodity hardware, which has limited computing power. This is impractical for applications requiring instant interaction. Conversely, lightweight RNNs often sacrifice precision for speed. Therefore, a new model architecture is urgently needed. It must balance high-precision prediction with strict low-latency requirements.

Spiking neural networks (SNNs) offer a promising alternative by processing information in an event-driven manner. Information is transmitted through discrete spike events rather than continuous values [9], making SNNs highly suitable for modeling sparse and asynchronous temporal data. Recent advancements in deep SNNs have successfully integrated attention mechanisms [10,11] and specialized temporal architectures [12,13]. Despite these inspiring results, existing SNN models face three fundamental challenges when applied to private vehicle parking prediction:

- Most existing SNN models are built upon standard Leaky Integrate-and-Fire (LIF) neurons with fixed decay parameters. Such neurons lack the flexibility to capture the heterogeneous inter-event intervals observed in mobility data (ranging from minutes to days). In event-driven processing, standard neurons decay by event counts alone, equating long parking durations with short stops and causing outdated historical states to persist unnecessarily.
- Many SNN models rely on rate-based spike counting. In latency-sensitive scenarios where the number of simulation steps is limited, spike-count-based readouts introduce severe quantization effects, significantly degrading the accuracy of continuous values prediction.
- Current SNN models often lack effective, event-driven mechanisms to selectively regulate information flow across channels based on external context (e.g., weather, holiday) without breaking the sparse computing paradigm.

To address these challenges, we propose a spiking neural network for private vehicle parking prediction named Spark. Our approach redesigns the core components of SNNs to enhance temporal modeling flexibility and prediction accuracy while maintaining high computational efficiency. The main contributions of this work are summarized as follows:

- To effectively handle the challenge of irregular intervals, we propose a Time-Adaptive Leaky Integrate-and-Fire (TA-LIF) neuron. Unlike standard neurons with fixed decay, TA-LIF employs a lookup table (LUT)-based mechanism with logarithmic discretization. This design explicitly couples neuronal memory decay with physical time gaps, effectively capturing long-term regularities across varying timescales without incurring the computational overhead of transcendental functions.
- To mitigate quantization errors in regression, we introduce an accumulate-based readout (ABR) strategy. It mitigates the precision bottleneck of discrete spike counting by utilizing the continuous accumulated response of the readout neurons as the final output. Furthermore, this approach unifies classification and regression representations, enabling high-precision duration prediction even under minimal simulation steps.
- To enable selective information regulation, we design a Spiking Contextual Gating (SCG) module. It constructs a lightweight gating strategy that modulates spiking information flow based on environmental context to avoid expensive computational costs. Specifically, it generates dynamic binary masks to perform selective feature filtering, functionally replacing heavy matrix multiplications with lightweight sparse masking.

## 2. Related Work

### 2.1. Deep Learning for Mobility Prediction

Private vehicle trajectories, as a type of human movement trajectory, exhibit non-linear, dynamic behavior with inherent high-order characteristics. Therefore, mining knowledge from these trajectories has been widely considered an effective method for mobility prediction. Early researchers often utilized statistical methods such as Markov chains [5] and matrix factorization to extract mobility patterns from historical trajectory datasets. However, these models are designed to capture immediate and pairwise relationships, which failed to handle complex and high-order dependencies inherent in private vehicle mobility behaviors. Later, researchers turned to deep neural networks to learn such high-order dependencies, achieving promising results. DeepMove [6] believed that multi-level periodicity in human mobility is very important, and designed a model based on RNN to capture it. DeepRP [7] considered regularity and preference as the most important movement features, and introduced a backtracking attention mechanism with RNN to explicitly model these two features for location prediction. GETNext [14] incorporated global transition patterns, user preferences, and time-aware category embeddings into a Transformer architecture to achieve a better spatial context modeling for prediction. DiffTraj [15] formulated trajectory generation as a denoising process and introduced spatial-temporal diffusion models for mobility prediction. iTransformer [16] applied a strategy of using attention and feed-forward networks on inverted dimensions, efficiently modeling multivariate correlations and achieving state-of-the-art predictive performance. Time-LSTM [17] introduced time gates to explicitly regulate information flow based on time intervals, mitigating the bias of uniform step assumptions. Furthermore, recent advances in continuous-time modeling, such as Neural Ordinary Differential Equations [18], modeled the hidden state evolution as a continuous function, theoretically offering superior handling of irregular intervals. Similarly, Neural Hawkes Processes [19] combined point processes with deep learning to capture the intensity of event occurrences. Recently, large language models (LLMs) have stood out among various models, bringing exciting confidence to mobile prediction. UrbanGPT [20] integrated a spatio-temporal instruction-tuning strategy into a spatio-temporal LLM, which enables it to comprehend complex spatio-temporal inter-dependencies and strong generalization capability.

Despite the impressive performance achieved by the above state-of-the-art artificial neural network (ANN) models, they generally suffer from high computational complexity. For instance, diffusion-based models require multiple iterative denoising steps and Transformer-based architectures exhibit a quadratic growth in computational complexity with respect to sequence length, resulting in substantial floating-point computation overhead. Continuous-time models typically rely on computationally intensive numerical solvers or dense attention mechanisms, posing significant challenges for deployment on resource-constrained vehicular edge devices. Consequently, state-of-the-art ANN models confront formidable challenges when deployed in resource-constrained environments such as vehicular edge devices.

### 2.2. Spiking Neural Networks for Spatio-Temporal Data

Unlike ANNs with heavy computation, spiking neural networks use discrete binary spikes and replace multiply–accumulate with accumulate operations, which offer a promising efficient approach for time series prediction. With surrogate gradient learning, SNNs are capable of complex spatio-temporal prediction. Early research focused primarily on mimicking biological synaptic plasticity; the learning rule of spike timing dependent plasticity was proposed for extracting repetitive spatio-temporal patterns [21]. Recent works integrated attention mechanisms into SNNs to pursue higher performance. For instance, STSA-SNN [10] incorporated self-attention into SNNs for capturing high-order dependencies without destroying asynchronous transmission. TCJA-SNN [11] fused a temporal-channel joint attention mechanism in spiking neural networks to achieve better performance. More recently, iSpikformer [12] fused temporal alignment, convolutional spike encoders, and counterparts of Transformers into a unified framework for capturing complex patterns in spatio-temporal data. TS-LIF [13] believed that long-term dependencies and multi-scale temporal dynamics are critical for spatio-temporal forecasting and proposed a temporal segment neuron with dual compartments (dendritic and somatic) model to capture multi-scale dynamics implicitly. In more recent work, NOS-Gate [22] has explored event-driven processing for gateway security. Although NOS-Gate shares the low-latency goal, it targets anomaly detection using a two-state dynamic specifically for queue management. In contrast, Spark adapts the SNN architecture for mobility prediction, necessitating a distinct design (TA-LIF and ABR) to handle the regression precision and long-term memory required for parking duration estimation.

Although these SNN models have achieved promising results, they may struggle with heterogeneous time intervals, regression precision, and contextual adaptability when facing mobility prediction. Additionally, some attention-based SNNs suffer from quadratic computational complexity regarding sequence length, which contradicts the low-latency and low-power requirements of edge-based mobility prediction systems.

## 3. Preliminaries

### 3.1. Problem Definition

We consider the problem of private vehicle parking prediction as a multi-objective sequence learning task driven by discrete events. Unlike dense GPS trajectory data, private vehicle parking behavior is characterized by sparse, non-uniform events.

**Definition 1** 
(Parking Event). *A parking event $e_{i}$ is defined as a tuple $e_{i}=\left(l_{i},\tau_{i}^{arr},\delta_{i},c_{i}\right)$, where $l_{i}\in L$ represents the discretized location ID (e.g., POI), $\tau_{i}^{arr}$ denotes the absolute arrival timestamp, $\delta_{i}\in R^{+}$ is the continuous parking duration, and $c_{i}$ represents external context vectors (e.g., weather conditions, holiday status).*

**Definition 2** 
(Event Sequence). *The history of a user u is represented as a chronologically ordered sequence of N parking events $S_{u}=\left\{e_{1},e_{2},\ldots ,e_{N}\right\}$.*

**Definition 3** 
(Physical Time Interval). *To explicitly capture the irregular temporal dynamics, we define the physical time interval $\Delta t_{i}$ between consecutive events $e_{i-1}$ and $e_{i}$ as the elapsed time since the previous arrival:*

(1) $$\Delta t_{i}=\tau_{i}^{arr}-\tau_{i-1}^{arr}$$

*Note that $\Delta t_{i}$ exhibits a heavy-tailed distribution, ranging from minutes (short commutes) to days (e.g., long-term parking followed by a new trip), which poses a significant challenge for fixed-decay neurons.*

**Problem 1** 
(Joint Location and Duration Prediction). *Given the historical sequence $S_{u}$, the goal is to simultaneously predict the next parking location $l_{N+1}$ (classification) and the corresponding parking duration $\delta_{N+1}$ (regression). This can be formulated as learning a mapping function $F_{\theta }$ parameterized by θ:*

(2) $$\left({\hat{l}}_{N+1},{\hat{\delta }}_{N+1}\right)=F_{\theta }\left(S_{u}\right)$$

### 3.2. Spiking Neuron Dynamics

The fundamental computing unit in our framework is the LIF neuron. In the discrete-time formulation suitable for neuromorphic simulation, the dynamics of a standard LIF neuron *i* at layer *l* and time step *t* are governed by:

(3) $$U_{i}\left[t\right]=\beta \left(U_{i}\left[t-1\right]-V_{th}\cdot s_{i}\left[t-1\right]\right)+\sum_{j}w_{ij}s_{j}\left[t\right]$$

(4) $$s_{i}\left[t\right]=\Theta \left(U_{i}\left[t\right]-V_{th}\right)$$

where $U_{i}\left[t\right]$ is the membrane potential, β∈(0, 1) is the decay factor, $V_{th}$ is the firing threshold, $w_{ij}$ is the synaptic weight from neuron *j* to neuron *i*, $s_{i}\left[t\right]\in \left\{0,\;1\right\}$ is the spike output, and Θ(·) is the Heaviside step function.

Standard LIF neurons are typically simulated with fixed discrete time steps. However, private vehicle parking events are often irregularly sampled in reality. Under a strict low-latency regime, each event is simulated with a fixed and small number of steps *T*, and the neuronal state transition is driven only by event counts rather than the actual physical interval Δt. As a result, the spiking neuron network may preserve outdated historical information after long physical intervals and fail to reset or weaken memories correctly when needed. This discrepancy motivates time-adaptive neuronal dynamics, detailed in Section 4.2.

## 4. Methodology

**Figure 1:**
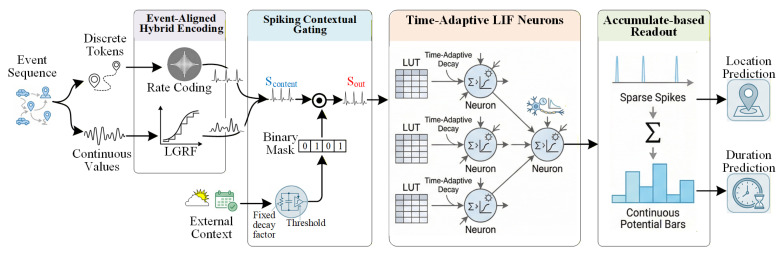
The overall framework of the proposed Spark.

In this section, we present an event-driven spiking neural network for private vehicle parking prediction, named Spark. As shown in Figure 1, the proposed architecture consists of four coupled components:

1. Event-Aligned Hybrid Encoding: It transforms heterogeneous raw data into spike-compatible formats.
2. Spiking Contextual Gating: It fuses environmental context (weather, holiday) to selectively modulate spatial preferences via lightweight gating.
3. TA-LIF Neuron: It dynamically adjusts neuronal memory based on physical time gaps to capture long-term regularity via the LUT-based mechanism.
4. Accumulate-based Readout: It performs high-precision regression and classification with minimized quantization error.

### 4.1. Event-Aligned Hybrid Encoding

Raw trajectory data comprises both discrete tokens (Location ID) and continuous values (Time, Duration). Standard direct coding is inefficient for this work, and thus we employ a hybrid strategy to preserve information density.

#### 4.1.1. Discrete Semantics

For categorical variables, we explicitly incorporate environmental context alongside the location information to capture external influences on mobility. The feature set comprises the following components: Location ID $l_{i}$, the discretized grid cell index representing the parking location; Weather Condition $w_{i}$, categorized into 8 types based on meteorological records (Sunny, Cloudy, Rainy, Snowy, Foggy, Storm, Hail, Frosty); and Calendar Status $h_{i}$, categorized into 3 types (Workday, Weekend, National Holiday). We utilize learnable embedding layers to map these discrete tokens into dense vectors $e_{loc},e_{wea},e_{hol}\in R^{D}$. To facilitate the Spiking Contextual Gating mechanism, the weather and holiday embeddings are fused (via element-wise addition) to form the context vector $e_{context}=e_{wea}+e_{hol}$. Subsequently, we employ a rate-coding encoder with a maximum firing probability $p_{max}$ to convert these continuous vectors into spike trains. For simulation steps t=1,…,T, the discrete context spike train $S_{disc}\left[t\right]$ is generated via a Bernoulli process:

(5) $$S_{disc}\left[t\right]∼\mathrm{Bernoulli}\left(\pi \right),\;\pi =p_{max}\cdot \sigma \left(e_{context}\right),\;p_{max}\in \left(0,\;1\right]$$

where σ(·) denotes the element-wise sigmoid function. The Bernoulli sampling is performed independently for each dimension of $\pi \in {\left[0,1\;\right]}^{D}$.

#### 4.1.2. Continuous Dynamics via Learnable Gaussian Receptive Fields

Direct injection of continuous values as constant injection currents often causes latency issues in spike-based encoding. We adopt learnable Gaussian receptive fields. We quantify a continuous scalar *v* into a population of $N_{\mathrm{enc}}$ neurons. The input current $I_{j}$ for the *j*-th neuron is:

(6) $$I_{j}\left(v\right)=\exp\left(-\frac{{\left(v-\mu_{j}\right)}^{2}}{2\sigma_{j}^{2}}\right)$$

where $\mu_{j}$ and $\sigma_{j}$ are learnable parameters initialized to cover the data distribution. This formulation ensures differentiability with respect to $\mu_{j}$ and $\sigma_{j}$, which is crucial for the backpropagation process during training.

It is worth noting that Equation (6) involves exponential calculations. We address the potential computational overhead during the inference phase by adopting a LUT strategy, consistent with our Time-Adaptive LIF design in Section 4.2. Since the parameters $\mu_{j}$ and $\sigma_{j}$ are frozen after training, the response of the *j*-th neuron becomes a static function of the input *v*. We pre-compute the Gaussian response curves into a discretized table $T_{\mathrm{in}}\in R^{B_{\mathrm{in}}\times N_{\mathrm{enc}}}$, where $B_{\mathrm{in}}$ is the number of quantization bins for the input range. During deployment on edge devices, the exact exponential calculation is replaced by a constant-time memory fetch operation:

(7) $$I_{j}\left(v\right)\approx T_{\mathrm{in}}\left[Q\left(v\right),j\right]$$

where Q(v) denotes the quantization function mapping the continuous value *v* to its nearest bin index. This strategy eliminates the runtime cost of transcendental functions in the encoding layer, ensuring that the temporal spike-based modeling path of Spark reduces floating-point arithmetic during inference. Specifically, the retrieved current $I_{j}\left(v\right)$ serves as a constant injection current to the population-based encoding LIF neurons over *T* simulation steps, where the continuous value is represented by the distributed Gaussian receptive-field responses rather than a direct single-channel current injection.

### 4.2. Time-Adaptive LIF Neuron

Human mobility exhibits regularity dependent on physical time rather than event steps. Standard SNNs use a fixed decay β, leading to temporal dynamics mismatch when the time gap Δt varies from minutes to days. We propose the TA-LIF neuron, which alleviates the discrepancy between fixed simulation steps and irregular physical time intervals.

#### 4.2.1. Dynamics

Let $U_{i}\left[t\right]$ be the membrane potential of neuron *i* at simulation step *t* (1≤t≤T). To explicitly model sequence dependencies, the initial potential of the current event inherits the final state of the previous event. The update rule incorporates a dynamic decay factor β(Δt):

(8) $$U_{i}\left[t\right]=\beta \left(\Delta t\right)\cdot \left(U_{i}\left[t-1\right]-V_{th}\cdot s_{i}\left[t-1\right]\right)+\sum_{j}w_{ij}s_{j}\left[t\right]$$

(9) $$s_{i}\left[t\right]=\Theta \left(U_{i}\left[t\right]-V_{th}\right)$$

where β(Δt) is an effective per-step decay factor, $V_{th}$ is the firing threshold, $w_{ij}$ is the synaptic weight connecting the pre-synaptic neuron *j* to the post-synaptic neuron *i*, and Θ(·) is the Heaviside step function. Given a fixed number of simulation steps *T* for each event, we use the continuous-time exponential decay as a guiding principle: the effective neuronal memory retention factor across *T* steps should decrease with larger Δt, i.e.,

(10) $$\beta {\left(\Delta t\right)}^{T}\approx \exp\left(-\frac{\Delta t}{\tau_{\mathrm{decay}}}\right)$$

where $\tau_{\mathrm{decay}}$ denotes a characteristic timescale. Importantly, we do not enforce this relation as a hard constraint; instead, β(Δt) is parameterized by a learnable LUT (Section 4.2.2) to flexibly fit dataset-specific temporal dynamics.

#### 4.2.2. Efficient Decay via Learnable LUT

A bottleneck in deploying time-aware SNNs is the calculation of the dynamic decay factor β(Δt). Previous approaches relying on exponential functions ($e^{-\Delta t/\tau_{\mathrm{decay}}}$) or rational approximations introduce expensive floating-point operations at every inference step, negating the computational sparsity advantage of SNNs. To mitigate this computational overhead and strictly align with the principles of efficient neuromorphic computing, we propose an LUT-based time-adaptive mechanism.

Instead of computing the decay value on-the-fly via complex functions, we discretize the continuous physical time interval Δt into *K* discrete bins and retrieve the decay factor from a learnable parameter vector $B\in {\left[0,\;1\right]}^{K}$. Therefore, the choice of discretization strategy is pivotal. Previous work shows that inter-event intervals in human mobility exhibit a scale-free property, primarily dominated by the heavy-tailed distribution of parking durations [4]. Thus, a linear mapping would inefficiently allocate the majority of bins to the sparse long-tail, while severely under-sampling the high-frequency events in short intervals.

To align the memory resolution with the information density of the data, we adopt a logarithmic discretization strategy. This approach ensures a constant relative resolution, providing fine-grained granularity for short stops and coarser granularity for long-duration parking. Formally, we discretize the continuous physical time interval Δt into bin index *k* using a logarithmic scale:

(11) $$k=\min\left(\max\left(\left\lfloor \log_{\alpha }\left(\Delta t+1\right)\right\rfloor ,0\right),K-1\right)$$

where α is the logarithmic base controlling the granularity growth. Instead of treating α as a heuristic hyperparameter, we determine it analytically to align the discretization resolution with the dataset statistics and the memory budget *K*.

Since the distribution of parking durations is heavy-tailed, relying on the absolute maximum value ($\Delta t_{\max}$) may lead to inefficient bin utilization, where most bins are allocated to rare, extremely long intervals. To ensure robustness against outliers, we define an effective upper bound $T_{eff}$ as the *p*-th percentile of the training time intervals (e.g., p=99). Any interval larger than $T_{eff}$ is clipped to this value. Consequently, the optimal base is computed as:

(12) $$\alpha ={\left(T_{eff}+1\right)}^{\frac{1}{K-1}}$$

This strategy ensures that the full resolution of the LUT is dedicated to the effective temporal range covering most of events, while rare long gaps are grouped into the final bin K−1. Since the neuronal membrane potential typically decays to its resting state over such long durations regardless of the exact decay factor, this clipping introduces negligible error while significantly enhancing granularity for the majority of samples.

This data-driven formulation guarantees that the model fully utilizes the allocated memory capacity. It provides the finest possible granularity for high-frequency short trips while maintaining the capability to capture long-tail events within B.

A potential theoretical concern with Equation (11) is the non-differentiability of the floor and clip operations. In this work, during the backward pass, the LUT mechanism functions strictly as a differentiable gather operation (similar to embedding layers), allowing gradients to flow directly into the selected parameter entry B[k]. During backpropagation, since the time interval Δt (and thus the index *k*) is fixed input data, the gradient does not need to propagate through the discretization step. Instead, the gradient of the loss L flows directly to the specific entry in the parameter vector B that was activated. Let $k_{e}$ denote the bin index computed from the physical interval $\Delta t_{e}$ of event *e*. Since $\Delta t_{e}$ (and thus $k_{e}$) is fixed input data, gradients do not propagate through the discretization step. Instead, the gradient is accumulated only on the activated LUT entry:

(13) $$\frac{\partial L}{\partial B[i]}=\sum_{e}\frac{\partial L}{\partial \beta (\Delta t_{e})}\cdot I\left(k_{e}=i\right)$$

where the summation runs over all events *e* in the mini-batch, and I(·) is the indicator function. This allows the decay profile B to be optimized end-to-end alongside synaptic weights, automatically learning the optimal temporal decay curve for specific mobility patterns without requiring gradients to flow through the logical operations.

From an algorithmic perspective, calculating the decay factor via LUT involves a constant-time memory lookup. For each event (and for each sample in a batch), the physical interval Δt is shared across neurons within the same layer, so the index computation and LUT fetch produce a scalar decay factor β(Δt) that can be broadcast to all neurons in that layer for that event. This avoids per-neuron transcendental-function evaluation and preserves the computational sparsity advantage of SNNs.

### 4.3. Spiking Contextual Gating

Individual mobility is highly context-sensitive [7]. To model this non-linear coupling while preserving the event-driven sparse computing pattern and reducing dense floating-point multiply–accumulate operations, we propose the SCG module.

Let $S_{content}\in {\left\{0,\;1\right\}}^{B\times T\times C}$ be the spike features from the main trajectory branch, and $S_{context}$ be the features from the context branch (e.g., Weather). Unlike previous soft-gating mechanisms or attention modules that rely on Sigmoid/Softmax functions, which require heavy multiply–accumulate operations, SCG generates a binary spike mask to filter features via a bitwise-implementable gating mechanism.

The context features are processed by a linear synaptic layer followed by an LIF neuron to generate the binary gating mask $M\left[t\right]\in {\left\{0,1\right\}}^{C}$. The dynamics of the gating LIF neurons follow a fixed decay factor to maintain temporal stability over the gating window:

(14) $$U_{gate}\left[t\right]=\beta_{gate}\left(U_{gate}\left[t-1\right]-V_{th}^{gate}M\left[t-1\right]\right)+W_{gate}S_{context}\left[t\right]+b_{gate}$$

(15) $$M\left[t\right]=\Theta (U_{gate}\left[t\right]-V_{th}^{gate})$$

where $\beta_{gate}$ is a fixed decay factor, $V_{th}^{gate}$ is the firing threshold, $W_{gate}$ and $b_{gate}$ denote the synaptic weight matrix and bias vector, respectively, and Θ(·) is the Heaviside step function. The feature fusion is performed via element-wise multiplication, which is functionally equivalent to a logical gating operation for binary spike representations. Since M[t]∈{0, 1} acts as a binary gate, features are passed only when M[t]=1, effectively filtering out context-irrelevant spatial features:

(16) $$S_{out}\left[t\right]=S_{content}\left[t\right]⊙M\left[t\right]$$

In implementation, since both $S_{\mathrm{content}}\left[t\right]$ and M[t] are binary, Equation (16) is functionally equivalent to a logical AND operation, thereby reducing unnecessary dense floating-point multiply–accumulate operations. To prevent persistent neuronal silence (where M[t] remains 0, blocking all flow), we initialize the bias of the gating LIF layer to a positive value, ensuring gates are initially open. The network then learns to selectively inhibit irrelevant features based on context, effectively acting as a learnable pruning mechanism.

It is worth noting that in GPU-based implementation, the mask M[t] acts as a logical filter (element-wise multiplication). However, on neuromorphic hardware or event-driven accelerators, a zero value in the binary mask physically prevents the generation of current or spikes for the corresponding channels. Consequently, the SCG module leads to actual skipped synaptic operations (SOPs) in deployment, directly translating the mask sparsity into energy savings (quantitative analysis is provided in Section 5.3).

### 4.4. Accumulate-Based Readout Strategy

A limitation of standard SNNs is the quantization error introduced by rate coding at the output layer (counting discrete spikes), which limits performance in regression tasks. To tackle this issue, and to unify the output representation for both classification and regression, we employ an ABR strategy for the final layer. To simultaneously handle the joint task of location classification and duration regression, we construct two parallel readout branches following this strategy.

Instead of firing spikes, the output layer consists of non-spiking neurons that aggregate the discrete spike inputs from the last hidden layer into continuous output values. Let $s_{last}\left[t\right]$ be the spike vector from the last hidden layer at time step *t*. The final output $U_{out}$, defined as the total accumulated response over the simulation window *T*, is calculated as:

(17) $$U_{out}\left[T\right]=W_{out}\cdot \sum_{t=1}^{T}s_{last}\left[t\right]+b_{out}$$

where $W_{out}$ and $b_{out}$ represent the learnable weight matrix and bias vector of the readout layer, respectively. This strategy mitigates the quantization constraints inherent to discrete spike counting by utilizing the continuous accumulated response $U_{out}\left[T\right]$, which is projected from the aggregated spike train for the final prediction.

For location prediction: The output potentials are directly utilized as logits for the Softmax function to compute the class probabilities ${\hat{p}}_{c}$.

For parking duration estimation: The scalar prediction ${\hat{\delta }}_{\log}$ is directly derived from the output potential and represents the log-transformed duration. During the inference phase, this value is projected back to the physical domain as the final predicted duration $\hat{\delta }$ via the inverse transformation:

(18) $$\hat{\delta }=\exp\left({\hat{\delta }}_{\log}\right)-1$$

By avoiding quantization errors inherent in spike counting, the ABR strategy ensures that the classification head also benefits from the fine-grained information preserved in the continuous accumulated values.

### 4.5. Training and Optimization

Training deep SNNs is non-trivial due to the non-differentiability of the spike generation function Θ(·). We employ a robust training pipeline to ensure convergence.

#### 4.5.1. Surrogate Gradient Learning

During the backward pass, we replace the derivative of the Heaviside function $\Theta^{\prime }\left(x\right)$ with a surrogate gradient function $\sigma^{\prime }\left(x\right)$. We use the derivative of the Arctan function, known for its stability:

(19) $$\frac{\partial s}{\partial u}\approx \frac{\gamma }{1+{\left(\gamma \cdot \left(u-V_{th}\right)\right)}^{2}}$$

where *u* represents the membrane potential, $V_{th}$ is the firing threshold, and γ determines the width of the gradient window. This allows gradients to flow through discrete spikes to update weights W and vector B in the LUT.

#### 4.5.2. Loss Function

We formulate the task as a multi-objective optimization problem. We treat the location prediction as a multi-class classification problem. The Cross-Entropy loss with LogSoftmax is employed to maximize the probability of the ground-truth location:

(20) $$L_{loc}=-\sum_{c=1}^{N_{class}}y_{c}\log\left({\hat{p}}_{c}\right)$$

where $N_{class}$ is the number of location categories, and $y_{c}$ is the binary indicator (0 or 1) for class *c* in the ground-truth label.

Parking duration follows a long-tail distribution. To be robust against outliers, we perform regression in the log-scale domain using the Smooth L1 Loss (Huber Loss):

(21) $$L_{dur}=\left\{\begin{array}{cc} 0.5{\left({\hat{\delta }}_{\log}-\delta_{\log}\right)}^{2}, & \mathrm{if}\;|{\hat{\delta }}_{\log}-\delta_{\log}|<1\\ |{\hat{\delta }}_{\log}-\delta_{\log}|-0.5, & \mathrm{otherwise} \end{array}\right.$$

where ${\hat{\delta }}_{\log}$ denotes the predicted log-transformed parking duration, and $\delta_{\log}=\ln\left(1+\delta_{gt}\right)$ represents the ground-truth log-transformed duration derived from the physical label $\delta_{gt}$.

To prevent network silence and maintain information flow, we add a firing rate regularization term $L_{reg}$ to encourage a healthy sparsity level.

(22) $$L_{reg}=\frac{1}{L}\sum_{l=1}^{L}{\left({\bar{s}}_{l}-r_{target}\right)}^{2}$$

where *L* denotes the total number of spiking layers, $r_{target}$ is the target firing rate hyperparameter, and ${\bar{s}}_{l}$ represents the mean firing rate of neurons in the *l*-th layer averaged over the batch and simulation steps. Therefore, the total loss is:

(23) $$L_{total}=L_{loc}+\lambda_{1}L_{dur}+\lambda_{2}L_{reg}$$

where $\lambda_{1}$ and $\lambda_{2}$ are hyperparameters balancing the contributions of the regression and regularization terms, respectively.

## 5. Experiments

To comprehensively evaluate the performance, efficiency, and robustness of the proposed Spark, we conducted extensive experiments on three large-scale real-world vehicle trajectory datasets.

### 5.1. Experimental Setup

To ensure the reproducibility and fairness of our evaluation, this section details the datasets, the baselines, and the specific implementation parameters.

#### 5.1.1. Datasets and Preprocessing

To evaluate the effectiveness of Spark in real-world scenarios, we utilize three private vehicle trajectory datasets including three major cities in China: Shanghai, Shenzhen, and Changsha, spanning from 1 January 2023 to 1 January 2024. These trajectory datasets are publicly available on GitHub (https://github.com/HunanUniversityZhuXiao/PrivateCarTrajectoryData, accessed on 15 October 2025). To strictly distinguish valid parking behaviors from transient traffic stops, we define a parking event based on the engine ignition status recorded by the On-Board Diagnostics system. Specifically, a trajectory segment is labeled as a parking event only if (1) the engine status transitions to “Ignition-OFF”; and (2) the dwell time exceeds a threshold of 15 min. This duration constraint effectively filters out noise such as traffic light waits, congestion, and short pick-up/drop-off stops, preventing label leakage where traffic pauses are misclassified as parking destinations. Geographically, we adopt a grid-based discretization strategy acting as a static clustering radius. We partition the geographical space of each city into orthogonal grid cells with a spatial resolution of $0.01^{{}^{\circ}}\times 0.01^{{}^{\circ}}$ (approximately 1km×1km). Each grid cell is assigned a unique location ID. To filter out transient stops or GPS noise, we only retain grid cells that contain at least 10 valid parking events. The parking duration is calculated as the time difference between the ignition-off and ignition-on events detected by the vehicle’s On-Board Diagnostics system. The summary of datasets is described in Table 1. For each dataset, we selected 80% of the sequences for training, 10% for validation, and 10% for testing.

Ethical Statement: The trajectory datasets used in this study were collected with user consent and have been fully anonymized. All personal identifiers were replaced with hashed unique IDs before our access. The collection and usage of data strictly adhere to the data release protocols of the providing organizations.

#### 5.1.2. Baselines

Although generative models and large language models have recently shown promise in mobility prediction, they incur massive computational overheads and high inference latency. Since the primary scope of this work is energy-efficient and real-time prediction for vehicular edge devices, massive generative models fall outside the feasible operating range of our target scenarios. Therefore, we exclude general-purpose LLMs from our baselines. However, to provide a rigorous performance benchmark, we include state-of-the-art time-series Transformers (despite their higher computational cost) alongside lightweight, edge-deployable architectures to ensure a comprehensive assessment of efficiency–accuracy trade-offs. We compare Spark against several competitive baselines.

- Markov [5]: A first-order Markov chain that calculates the transition probabilities between regions based on historical trajectories.
- DeepRP [7]: A GRU-based model with specific regularizers for location prediction.
- iTransformer [16]: The representative Transformer architecture.
- Time-LSTM [17]: A variant of LSTM to model time intervals explicitly.
- ODE-RNN [18]: A continuous-time RNN model that uses Neural ODEs to model the hidden state evolution between observations.
- THP [19]: It incorporates the intensity modeling of Hawkes Processes into a Transformer architecture to capture event dynamics.
- TCJA-SNN [11]: It proposes temporal-channel joint attention to recalibrate features in both dimensions cooperatively.
- iSpikformer [12]: A fully spiking Transformer adapted for time-series forecasting, utilizing spike-driven self-attention.
- TS-LIF [13]: A temporal segment LIF neuron with dual compartments designed to capture multi-scale temporal dynamics.

#### 5.1.3. Implementation Details

Several baselines were originally designed for next-location prediction. To ensure a comparison on the joint task of location and duration prediction, we standardized the output architecture across all learnable baselines. Specifically, we extended the final hidden layer of each baseline with two parallel branches: (1) a classification head using a linear projection followed by Softmax for location probabilities; and (2) a regression head using a linear projection for ANNs and a spike-count readout for standard SNN baselines. To ensure a fair comparison and quantify the contribution of our architecture independent of the readout layer, we explicitly analyze the impact of equipping baselines with the accumulate-based readout strategy. The detailed performance comparison and impact analysis are presented in Section 5.5.3. For statistical baselines like Markov, parking duration is predicted based on the historical average duration of the corresponding transition.

The proposed model Spark is implemented using PyTorch 2.7.1 and the SpikingJelly 0.0.0.0.14 [23] neuromorphic framework. The network architecture comprises an encoding layer with 128 neurons, followed by two TA-LIF recurrent layers with 256 neurons each. The hidden dimension of the SCG module is set to 256. To ensure effective gradient propagation through discrete spikes, we utilize the ArcTan surrogate gradient function (Equation (19)) with a width factor γ=2.0. For weight initialization, we employ the Kaiming Uniform method to maintain signal variance across layers, while the learnable decay parameters in the LUT (K=64) are initialized to 0.5 to start with a neutral temporal memory. We optimize the model using the AdamW optimizer with a cosine annealing scheduler, an initial learning rate of $1\times 10^{-3}$, and a batch size of 64. The hyperparameters for the multi-objective loss function are set to $\lambda_{1}=1.0$ and $\lambda_{2}=0.1$, with a target firing rate $r_{target}=0.05$ to enforce sparsity. To prevent overfitting, we apply a dropout rate of 0.2 within the SCG module and utilize an early stopping strategy based on validation loss with a patience of 20 epochs. Consistent with standard time-series evaluation, we strictly respect the chronological order for data splitting to prevent future information leakage. All experiments are repeated with five different random seeds, and we report the mean results to ensure statistical reliability.

A trade-off in SNNs exists between inference latency and information capacity, governed by the simulation time steps *T*. While increasing *T* typically improves the resolution of rate-coded SNNs, it linearly escalates the computational cost and latency, violating the constraints of real-time vehicular edge computing. Although different SNN architectures may achieve their optimal performance under different simulation step settings, we adopt a unified low-latency configuration (*T* = 4) to reflect realistic edge deployment constraints and to avoid introducing additional hyperparameter bias. This setting represents a strict low-latency regime. However, we recognize that this might put rate-coded baselines at a disadvantage. Therefore, we provide a comprehensive sensitivity analysis on *T* in Section 5.6 to demonstrate how different architectures behave under varying latency budgets.

#### 5.1.4. Evaluation Metrics

We comprehensively evaluate the model using the following metrics.

(1) Location Prediction Metrics: Since parking location prediction is a ranking problem, we employ Top-k Accuracy (Acc@k). Given *M* test samples, let $l_{i}$ be the ground truth location ID and $P_{i}$ be the predicted probability vector:

(24) $$\mathrm{Acc}\text{@}k=\frac{1}{M}\sum_{i=1}^{M}I\left(l_{i}\in {\mathrm{top}}_{k}\left(P_{i}\right)\right)$$

We report Acc@1 (Strict Precision) and Acc@5, with Acc@5 being particularly critical for intelligent vehicle services, as the ranking of the top five potential destinations aligns more closely with the actual workflow of recommendation systems.

(2) Duration Prediction Metrics: Parking duration follows a long-tail distribution. To evaluate performance in real-world units, we apply the inverse logarithmic transformation to the model predictions. Let $\delta_{i}$ denote the ground truth duration in physical hours, and ${\hat{\delta }}_{\log,i}$ be the model’s direct log-scale output. The final predicted physical duration ${\hat{\delta }}_{i}$ is obtained via ${\hat{\delta }}_{i}=\exp\left({\hat{\delta }}_{\log,i}\right)-1$. We calculate Mean Absolute Error (MAE) and Root Mean Squared Error (RMSE) in the physical domain as follows:

(25) $$MAE=\frac{1}{M}\sum_{i=1}^{M}\left|\delta_{i}-{\hat{\delta }}_{i}\right|$$

(26) $$RMSE=\sqrt{\frac{1}{M}\sum_{i=1}^{M}{\left(\delta_{i}-{\hat{\delta }}_{i}\right)}^{2}}$$

MAE provides a robust estimation for the majority of frequent, short-term stops, while RMSE imposes heavier penalties on large errors, serving as a critical indicator of the model’s ability to capture long-term dependencies.

### 5.2. Overall Performance Comparison

To demonstrate the superiority of Spark in handling complex mobility patterns, we present a comprehensive quantitative comparison against state-of-the-art baselines on both next-location prediction and parking duration estimation tasks. Table 2 presents the performance comparison results, from which we can obtain the following observations:

- Spark achieves superior performance compared to statistical, RNN, and continuous-time baselines. The Markov baseline yields the worst results on both tasks, as first-order transitions are insufficient to capture the complex dependencies in private vehicle mobility. DeepRP outperforms the statistical Markov model, verifying the capability of RNNs to capture sequential dependencies. However, DeepRP lags behind Time-LSTM, which confirms that explicitly incorporating time intervals into the memory gating mechanism is essential for modeling irregular mobility sequences, where uniform time steps are an invalid assumption. ODE-RNN and THP achieve highly competitive results, which verifies that modeling the continuous evolution of hidden states or event intensities is theoretically well-suited for asynchronous parking events. Spark outperforms all the aforementioned baselines, with this improvement attributed to the robustness of the proposed TA-LIF neuron. Spark’s LUT-based decay mechanism acts as a form of discrete regularization. It learns a stable, non-parametric decay profile that captures the underlying temporal laws without being overly sensitive to stochastic noise in the raw timestamps.
- Among the SNN baselines, Spark achieves the best performance in both tasks. Specifically, existing SNN baselines (e.g., TCJA-SNN and TS-LIF) rely on fixed decay factors, which result in inadequate decay of membrane potential during long parking intervals and thus lead to the retention of irrelevant historical noise. In contrast, Spark employs a learnable LUT mechanism to explicitly couple the leakage rate with physical time. This unique design enables Spark to adaptively reset neuronal states after long gaps and filter out outdated context information. Furthermore, Spark introduces the accumulate-based readout strategy, which integrates discrete spike trains into continuous accumulated values in the final non-spiking layer, instead of relying on discrete spike count statistics. This approach allows Spark to generate high-precision continuous outputs, which is particularly advantageous for the parking duration estimation task.
- Spark achieves results comparable to iTransformer with a mere 0.3% performance relative gap in Top-1 Accuracy and an MAE relative gap within 1.5%. This validates that Spark can capture complex spatial dependencies as effectively as global self-attention mechanisms.
- Spark exhibits strong generalization and avoids overfitting in prediction. It achieves consistent excellent performance on three datasets using identical hyperparameters, indicating it learns transferable temporal patterns rather than fitting local geography. Additionally, its event-driven sparsity serves as a form of intrinsic regularization analogous to dynamic dropout, which prevents over-reliance on specific features, and the synchronous decrease in training and validation losses under a strict chronological train–test split confirms that its strong generalization arises from genuine temporal laws rather than data leakage or overfitting.

### 5.3. Efficiency Analysis

After verifying the superior predictive accuracy, we further evaluate the computational efficiency and energy consumption to validate suitability for vehicular edge devices. We measure the inference speed of all models on a standard industrial personal computer acting as an edge surrogate. The device is equipped with an Intel Core i5-1135G7 @ 2.40 GHz Processor and 16 GB RAM. This platform operates under a typical thermal design power of 15 W, which is representative of the power envelopes found in smart cockpit controllers or on-board units.

**Figure 2:**
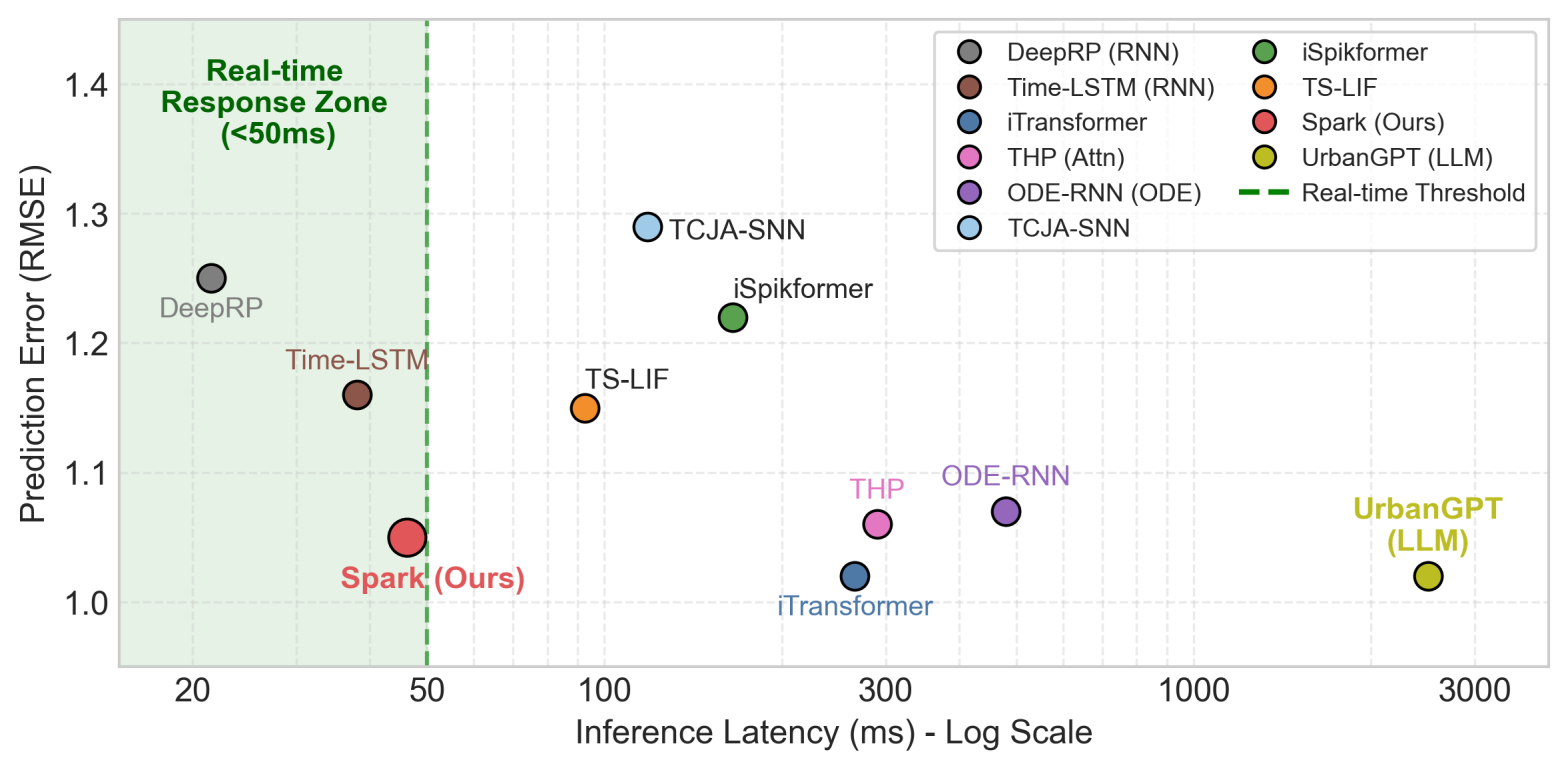
Parking duration prediction error (RMSE) vs. real-time latency trade-off.

As the performance metrics showed nearly identical results, we present only the result of RMSE and inference latency for brevity. Figure 2 illustrates the trade-off between prediction error (RMSE) and inference latency. The results reveal several critical insights:

- DeepRP achieves the lowest inference latency of 23.5 ms due to its simple, single-step recurrence dynamics. However, its limited capacity to model long-term dependencies results in a higher prediction error.
- While ODE-RNN achieves high prediction accuracy, it exhibits the highest latency (>400 ms per sequence). This is primarily due to the numerical differential equation solver, which requires multiple iterative evaluations to integrate the hidden state over time. Similarly, THP suffers from quadratic complexity due to its attention mechanism. Such high latency exceeds the response time requirements for seamless user interaction in smart cockpits.
- iTransformer provides the highest precision but at a prohibitive cost of 265.8 ms per sequence. This latency exceeds the threshold typically required for seamless real-time interaction in vehicular systems.
- UrbanGPT achieves the lowest RMSE of 1.02, benefiting from its strong pre-trained semantic reasoning and deep contextual understanding. However, its inference latency exceeds 2500 ms on edge CPUs, which is two orders of magnitude slower than the real-time constraint. This qualitative comparison highlights that while LLMs excel in contextual understanding, they remain computationally infeasible for latency-sensitive smart cockpit loops.
- Spark achieves an inference latency of 46.2 ms, primarily due to its event-driven architecture with recurrent neuronal dynamics, which avoids the heavy matrix computations of attention mechanisms used in SNN Transformers, while maintaining a low simulation step count. Although this is approximately 2× the latency of the RNN baseline, it remains within the Real-Time Response Zone in our setting (typically <50 ms). The results indicate that Spark achieves a favorable trade-off between accuracy and latency.

To further validate the efficiency of Spark, we evaluated the model using hardware-oriented metrics including synaptic operations, theoretical energy consumption, model capacity, and runtime memory footprint. To demonstrate that the performance gains stem from the proposed architectural mechanisms rather than an increase in model capacity, we compared Spark with representative baselines across all categories in terms of parameter counts and activation density.

Table 3 presents the efficiency comparison on the Shanghai dataset. We calculated the memory footprint based on a batch size of 64 during inference. The activation density denotes the percentage of non-zero elements in the feature maps averaged across layers. For ANNs, this is typically 100% due to continuous value propagation, whereas for SNNs, it represents the average spike firing rate.

The results in Table 3 reveal several critical insights regarding the trade-off between capacity and performance:

- Spark maintains a lightweight parameter scale (1.28 M), which is comparable to the GRU-based DeepRP (1.25 M) and significantly smaller than the attention-based iTransformer (3.42 M). This confirms that Spark’s superior prediction accuracy does not rely on over-parameterization but stems from the effective modeling of irregular temporal dynamics via the TA-LIF neurons. Furthermore, while ANN baselines exhibit 100% activation density due to dense matrix multiplication, Spark’s event-driven nature (4.8% density) drastically reduces the energy footprint.
- Although ODE-RNNs are theoretically adept at handling irregular intervals, they incur prohibitive computational costs (52.0 M FLOPs) due to the iterative nature of numerical solvers. In contrast, Spark captures similar continuous dynamics via the lookup table (LUT) mechanism with negligible overhead, achieving a 700× reduction in energy consumption.
- Among the spiking baselines, Spark achieves the lowest operation count and memory usage. For instance, iSpikformer, while powerful, inherits the heavy parameterization of Transformers (3.15 M params) and higher memory usage (42.0 MB). Spark outperforms these SNN baselines with a leaner architecture, validating the efficiency of the proposed SCG masking and accumulate-based readout strategies.

### 5.4. Ablation Study

To dissect the contribution of each component in Spark, we conduct a component-wise ablation study on three datasets. For simplicity, we only report the results with the Shanghai dataset in Table 4, as the results on other datasets are similar.

Table 4 contains the following observations:

- Effectiveness of TA-LIF: Replacing the standard fixed-decay LIF with our Time-Adaptive LIF resulted in a 7.9% improvement in the Acc@1 metric and 13.9% reduction in RMSE. This validates that TA-LIF can effectively address the temporal dynamics mismatch between simulation steps and physical time.
- Effectiveness of SCG: Introducing the SCG module improves accuracy by an additional 2.9% and reduces RMSE from 1.42 to 1.26. This result indicates that the auxiliary context provides crucial gating signals that effectively filter spatial preferences rather than acting as random noise.
- Effectiveness of ABR: Incorporating the ABR strategy reduces RMSE from 1.65 to 1.35. This confirms our hypothesis that rate-based spike counting induces severe quantization errors in regression tasks. The continuous-valued accumulation effectively mitigates this limitation.

### 5.5. Analysis of Modeling Mechanisms

To provide a comprehensive understanding of Spark’s internal mechanisms, this section performs a deep dive into the rationale behind our key design choices. Specifically, we investigate the optimality of time discretization strategies and the robust determination of the logarithmic base, validate the superiority of the uniform accumulate-based readout against leaky variants, and verify the model’s stability in handling irregular temporal dynamics across varying time intervals.

#### 5.5.1. Analysis of Time Discretization Strategies

To rigorously justify the adoption of the logarithmic discretization (Equation (11)) over other potential mapping strategies, we conducted a comparative analysis focusing on the statistical alignment between the binning mechanism and the intrinsic distribution of mobility data. Given that vehicle parking durations follow a heavy-tailed Power-Law distribution [4,25]; thus, standard uniform sampling is theoretically suboptimal. We compared our approach against two baselines:

1. Linear Binning: Dividing the maximum time window into equal-width bins.
2. Quantile Binning: Using adaptive boundaries such that each bin contains an equal number of training samples (theoretical maximum entropy).

**Figure 3:**
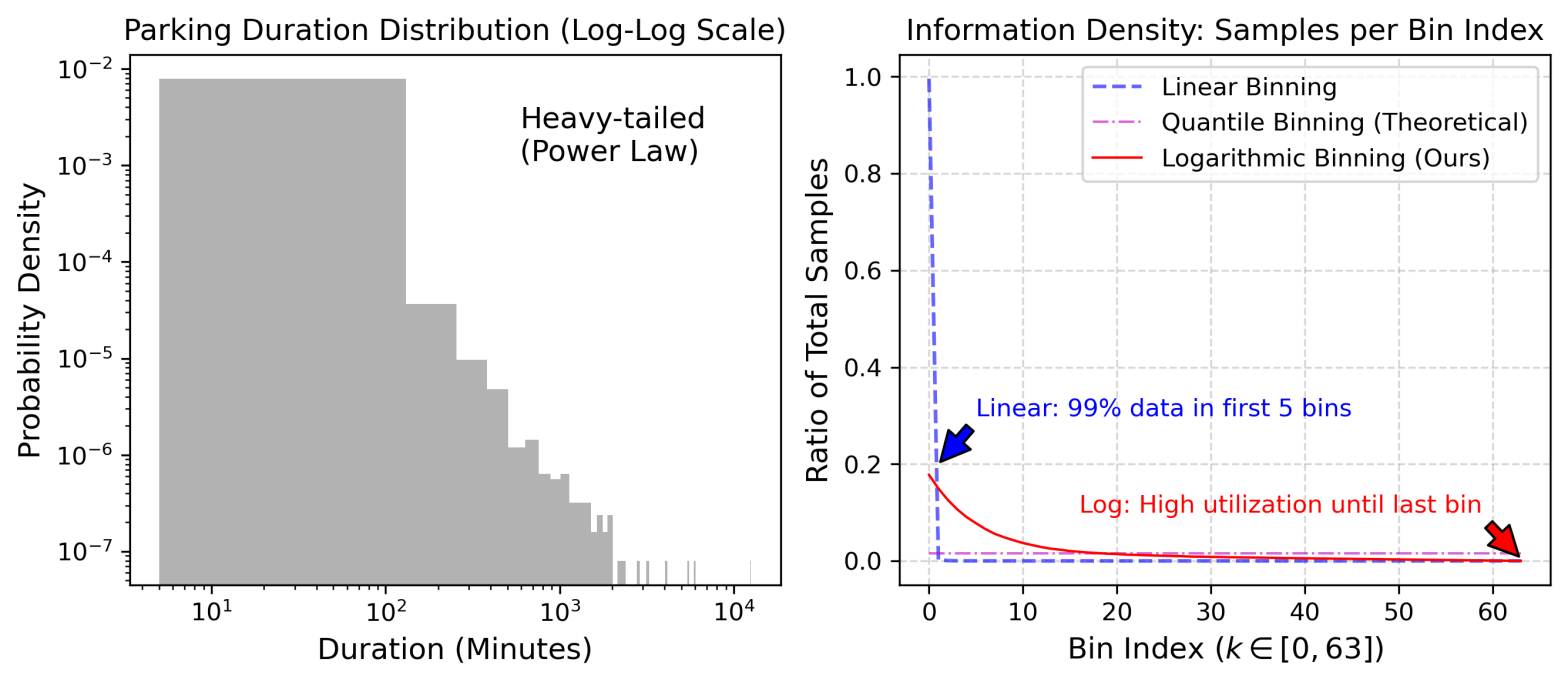
Impact of discretization strategy on information density. (**Left**) The parking duration distribution on a Log–Log scale confirms the scale-free, heavy-tailed nature of the dataset. (**Right**) Comparison of sample allocation across K=64 bins.

As illustrated in Figure 3, the choice of discretization significantly impacts the information density of the learnable parameters. Linear Binning suffers from a severe mismatch with the data distribution. Due to the heavy tail, the vast majority of parking events (short durations) are compressed into the first few bins, leaving the remaining bins (long durations) sparsely populated. This leads to sparse utilization in the LUT, where parameters corresponding to long-term dynamics are rarely accessed or updated due to a lack of training gradients.

In contrast, our logarithmic strategy exhibits a significantly more balanced bin utilization profile compared to linear binning, offering a trade-off that is closer to the information density of the ideal Quantile method while maintaining relative temporal resolution. By exponentially increasing the bin width, it maintains a constant relative resolution (Δt/t≈c). This ensures that the model allocates sufficient memory capacity to capture minute-level dynamics in short intervals while covering multi-day dependencies without exploding the memory budget.

From a computational perspective, although Quantile Binning offers the theoretical maximum entropy, it necessitates storing *K* floating-point boundary values and performing a binary search for every event during inference, leading to a complexity of O(logK). Our logarithmic approach achieves near-optimal statistical precision (balancing sample counts across bins) but utilizes a strictly O(1) mapping mechanism via efficient arithmetic operations, avoiding iterative search. This confirms that the proposed LUT-based logarithmic decay represents an effective engineering trade-off between statistical precision and the rigorous low-latency requirements of edge hardware.

#### 5.5.2. Analysis of Logarithmic Base Strategies

As discussed in Section 4.2.2, determining the logarithmic base α (Equation (12)) using the absolute maximum interval can be sensitive to outliers. To validate the effectiveness of our percentile-based truncation, we conducted a sensitivity analysis on the Shanghai dataset by varying the truncation threshold p∈{100%,99.9%,99%,95%}. Note that p=100% corresponds to using the raw maximum value.

**Figure 4:**
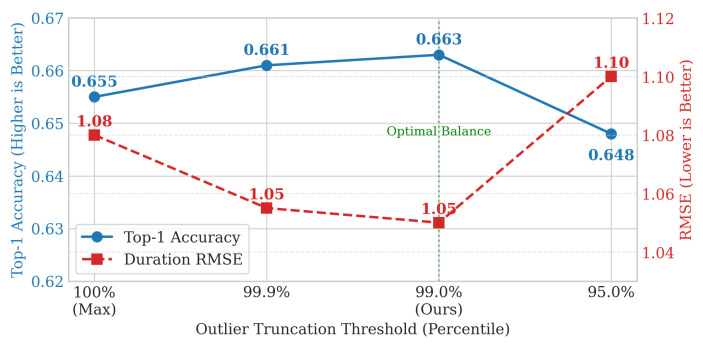
Sensitivity analysis of the outlier truncation threshold *p* on the Shanghai dataset.

The results are presented in Figure 4. We observe that:

- Using the raw maximum (100%) yields suboptimal performance (RMSE 1.08), this is because heavy-tailed outliers distort the bin allocation, leaving short-term intervals under-represented. Truncating at the 99th percentile significantly improves performance (RMSE 1.05), as it refines the granularity for the majority of data.
- Aggressively truncating at the 95th percentile degrades performance (RMSE rises to 1.10). This indicates that the “rare long gaps” (the top 1–5%) still contain valuable information regarding long-term periodicity (e.g., weekly or monthly patterns).

Therefore, selecting the 99th percentile acts as an optimal robust estimator, filtering out noise without harming the model’s ability to capture long-range dependencies. This strategy is adopted for all main results in Table 2.

#### 5.5.3. Analysis of Accumulate-Based Readout Strategies

To clarify the mechanism of the proposed accumulate-based readout (ABR) and investigate whether introducing temporal weighting improves performance, we conducted a comparative study. Technically, ABR functions as a uniform integrator with a decay factor α=1.0, treating spikes at all time steps equally. We compared this against a Leaky Readout strategy, which applies a temporal decay α<1.0 to the accumulated potential (i.e., U[t]=αU[t−1]+s[t]), thereby assigning higher weights to more recent spikes.

Table 5 summarizes the results on the Shanghai dataset. The comparison reveals that the uniform ABR strategy outperforms leaky variants. Specifically:

- With a strong decay (α=0.5), the RMSE degrades significantly to 1.22. In our low-latency setting (T=4), temporal decay excessively suppresses early spikes (e.g., a spike at t=1 contributes only 0.125× to the final output), reducing the effective signal magnitude and dynamic range required for high-precision regression.
- The mild decay (α=0.9) also yields suboptimal results (RMSE 1.11). Since the temporal dependencies are already explicitly captured by the preceding TA-LIF neurons, introducing additional temporal filtering at the readout layer creates redundancy and disrupts the learned representation. Thus, the uniform integration of ABR proves to be the robust approach for minimizing residual quantization errors.

To address the concern that the superior performance of Spark might stem solely from the continuous-valued ABR strategy rather than the temporal modeling capability of TA-LIF, we conducted a controlled experiment. We equipped representative SNN baselines (TCJA-SNN and TS-LIF) with the same accumulate-based readout (or leaky non-spiking readout) used in Spark and compared their regression performance (RMSE) against their standard rate-coding versions.

As shown in Table 6, replacing rate coding with ABR indeed reduces the prediction error for baselines, improving the RMSE of TCJA-SNN from 1.29 to 1.18 and TS-LIF from 1.15 to 1.10. This validates that the ABR strategy is indeed a generic optimization for low-latency SNN regression. However, strictly comparing the best-performing baseline (TS-LIF with ABR, RMSE 1.10) against Spark (RMSE 1.05), our model still maintains a clear lead. This confirms that while the readout layer is a contributing factor, the core advantage of Spark lies in the TA-LIF neuron’s ability to model irregular temporal intervals—a capability that fixed-decay baselines cannot achieve even with an improved readout.

#### 5.5.4. Analysis of Handling Irregular Temporal Dynamics

**Figure 5:**
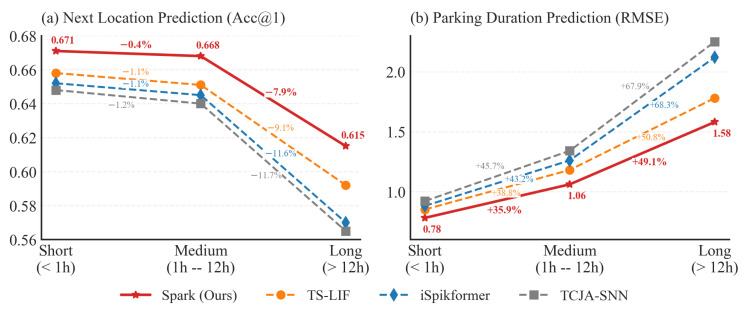
Performance variation across different time intervals.

To evaluate the model’s stability under irregular temporal sampling, we categorize the test samples into three groups based on the time gap Δt: Short (<1 h), Medium (1 h–12 h), and Long (>12 h). We only discuss the results on the Shanghai dataset for brevity, as experiments on other datasets are similar. Figure 5 explicitly details the performance variations for both next-location prediction (Acc@1) and parking duration estimation (RMSE).

As illustrated in Figure 5, two observations can be obtained:

- With the increase of time interval, the performance of all models is obviously decreased. This is consistent with the inherent increase in entropy in human mobility, where long-term behaviors exhibit higher randomness compared to short-term transitions. As the time interval increases, the uncertainty of behavior also increases greatly, making it more difficult to predict.
- Compared with the baselines, Spark’s performance in both tasks was least affected by time interval duration compared to the baselines. As the time interval increased from short to long, the performance of the baselines in both tasks showed a significant decline. The reason for this phenomenon is that the memory decay in the baselines depends solely on event counts, which cannot adapt to the dynamic variations in physical time intervals. Consequently, the networks retain noisy historical context information, which subsequently impacts subsequent task performance. Thanks to the TA-LIF neurons and LUT-based mechanism, Spark explicitly links neuronal leak rates to physical time intervals, dynamically adjusting information retention levels based on physical time gaps. In this way, Spark can effectively reset the state of neurons even after a long time interval, which not only reduces the interference of invalid noise, but also ensures the effective processing of the current task information by the network.

### 5.6. General Parameter Sensitivity

To further investigate the boundary conditions of the proposed mechanisms, we conduct a comprehensive sensitivity analysis on several critical hyperparameters: the simulation time steps, the LUT size, the learning rate, the weight decay, the target firing rate, and the spatial discretization.

#### 5.6.1. Sensitivity to Simulation Steps *T*

**Figure 6:**
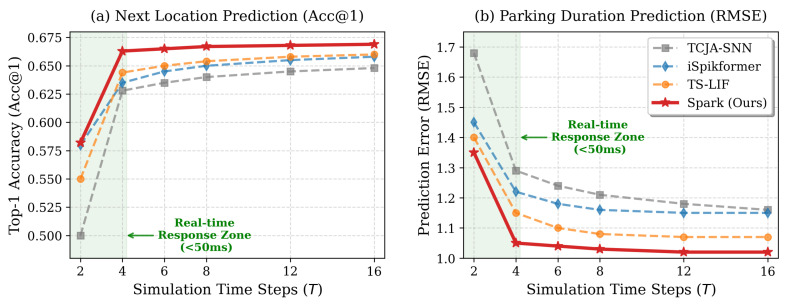
Performance sensitivity analysis under varying simulation time steps *T*. The green shaded area indicates the Real-Time Response Zone suitable for vehicular edge interaction.

In spiking neural networks, the number of simulation time steps *T* is the decisive factor regarding inference latency and information capacity. A fundamental limitation in conventional SNNs is the reliance on rate-based readout, where the precision is theoretically bounded by the discrete nature of spike counts (i.e., quantization error ∝1/T). To strictly evaluate the model’s capability under edge computing constraints, we compare Spark against state-of-the-art baselines across a range of time steps T∈{2,4,6,8,12,16}. For brevity, we only discuss the results for both classification (Acc@1) and regression (RMSE) on the Shanghai dataset results in Figure 6, as experiments on other datasets with all metrics are similar.

Figure 6 illustrates the following observations:

- All models suffer from significant performance degradation when *T* is restricted to 2. This phenomenon is primarily caused by the physical constraint of signal propagation depth in deep SNNs. Spikes require a minimum temporal window to propagate through multiple layers, and extremely short windows prevent the recurrent dynamics from effectively integrating historical context.
- Spark demonstrates the fastest convergence capability. When *T* increases to 4, Spark significantly outperforms baselines, reducing RMSE to 1.05 and achieving a Top-1 Accuracy of 0.663. This advantage stems from the proposed accumulate-based readout strategy. Unlike rate-coded baselines that require larger *T* to reduce quantization error for continuous regression, Spark leverages the continuous accumulated membrane potential in the final layer. This allows Spark to achieve high-precision regression and classification even under limited simulation steps, satisfying the Real-Time Response Zone requirements (<50 ms) without compromising accuracy.
- As *T* further increases from 6 to 16, the performance of rate-coded baselines gradually improves, narrowing the gap with Spark. For instance, iSpikformer achieves an RMSE of 1.10 at T=16, approaching Spark’s performance. This confirms that with sufficient latency budget, rate coding can eventually approximate high-precision values. However, Spark’s performance stabilizes after T=4, suggesting that the remaining error is dominated by the inherent aleatoric uncertainty of human mobility rather than model representational capacity. While increasing *T* yields marginal gains for baselines, it linearly escalates inference latency and energy consumption. Spark maintains its optimal performance–efficiency balance at T=4, whereas baselines require 3×∼4× more computational resources to reach comparable accuracy. Therefore, Spark remains the superior choice for resource-constrained vehicular edge scenarios.

#### 5.6.2. Sensitivity to LUT Size *K*

**Figure 7:**
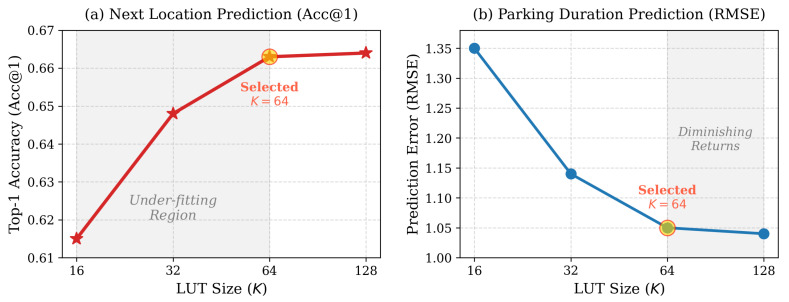
Impact of LUT Size *K* on model performance. The highlighted marker indicates the selected configuration (K=64).

The LUT size *K* is a pivotal hyperparameter governing the resolution of the time-adaptive decay mechanism. It determines how finely the continuous physical time intervals Δt are discretized into learnable decay factors. To identify the optimal configuration, we conducted a sensitivity analysis across all datasets with T=4 fixed, varying K∈{16,32,64,128}. The results across all datasets are largely similar, so for brevity, we present only the results for the Shanghai dataset. The joint performance impact on location prediction (Acc@1) and parking duration estimation (RMSE) is illustrated in Figure 7.

The results illustrated in Figure 7 indicate three observations:

- Setting a small *K* leads to suboptimal performance in both metrics. Specifically, at K=16, Acc@1 is suppressed at 0.615, and RMSE remains high at 1.35. This is caused by severe “quantization aliasing” under the logarithmic mapping strategy, where distinct physical intervals are mapped to the same decay parameter. This ambiguity forces the neuron to learn an averaged decay rate that fits neither scenario effectively, resulting in the loss of fine-grained temporal context required for both classification and regression.
- As *K* increases to 64, we observe a rapid performance gain, with Acc@1 reaching 0.659 and RMSE dropping to 1.09. At this resolution, the LUT provides sufficient granularity to distinguish subtle temporal patterns, such as decoupling short-term interactions from long-term periodicities. The TA-LIF neuron successfully matches precise leakage rates to varying time gaps, preserving user spatial preferences while minimizing regression errors.
- Further increasing *K* to 128 yields negligible marginal improvements (Acc@1 ≈ 0.660, RMSE ≈ 1.08). This saturation suggests that the temporal information gain has reached its limit. Theoretically, as *K* becomes large, the discretization granularity becomes excessively fine relative to the data density. Under a finite training dataset, this results in sparse activations for specific bins in the learnable vector B, preventing sufficient gradient updates. Moreover, the remaining error is largely attributed to the aleatoric uncertainty inherent in human mobility. Consequently, we select K=64 as the default configuration to balance model complexity and predictive accuracy.

#### 5.6.3. Sensitivity to Optimization Parameters

**Figure 8:**
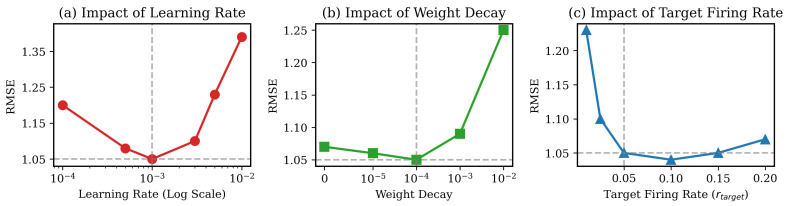
Sensitivity analysis of key hyperparameters on the Shanghai dataset. (**a**) Learning rate; (**b**) weight decay; (**c**) target firing rate. The dashed gray lines indicate the default settings used in our main experiments.

We further investigate the impact of the learning rate, the weight decay, and the target firing rate. All experiments were performed on the Shanghai dataset with simulation steps T=4. The results illustrated in Figure 8 indicate three observations.

- Learning Rate (η): As observed in Figure 8a, the model achieves optimal convergence with $\eta \approx 1\times 10^{-3}$. A smaller learning rate (e.g., $1\times 10^{-4}$) leads to under-fitting within the fixed training epochs, while an aggressive rate ($>5\times 10^{-3}$) destabilizes the surrogate gradient optimization, causing loss oscillation. This suggests that $1\times 10^{-3}$ effectively balances convergence speed and stability.
- Weight Decay: Figure 8b indicates that Spark is relatively robust to weight decay variations in the range $\left[0,1\times 10^{-3}\right]$. This stability can be attributed to the inherent regularization effects of the discrete spike generation and the dropout mechanism in the SCG module. We select $1\times 10^{-4}$ to prevent weight explosion without suppressing the representational capacity required for long-tail regression.
- Target Firing Rate ($r_{target}$): The choice of $r_{target}$ presents a critical trade-off between information capacity and sparsity. As shown in Figure 8c, an extremely low rate (<0.02) results in performance degradation due to vanishing gradients—neurons remain silent and fail to propagate error signals through time. Conversely, increasing $r_{target}$ beyond 0.1 yields negligible accuracy gains but significantly increases energy consumption. Consequently, we set $r_{target}=0.05$ to maximize energy efficiency while maintaining sufficient information flow for high-precision prediction.

#### 5.6.4. Sensitivity to Spatial Discretization

The choice of spatial discretization scheme (i.e., grid size) involves a trade-off between prediction precision and task difficulty. To investigate how the definition of “location” affects model performance, we conducted a sensitivity analysis on the Shanghai dataset by varying the grid resolution across three levels: Fine ($0.005^{{}^{\circ}}\approx 500\;m$), Standard ($0.01^{{}^{\circ}}\approx 1\;\mathrm{km}$), and Coarse ($0.02^{{}^{\circ}}\approx 2\;\mathrm{km}$).

**Figure 9:**
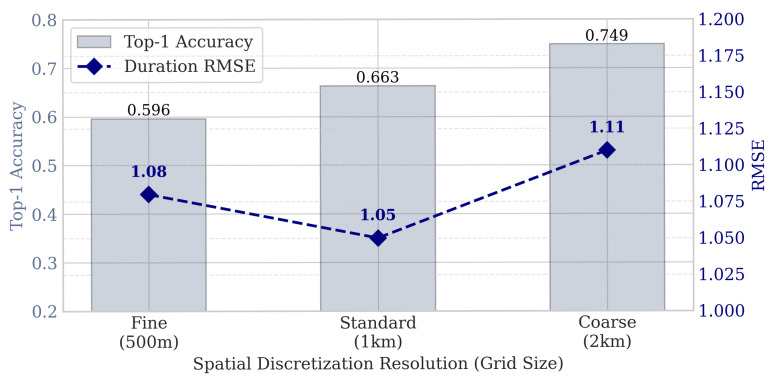
Impact of spatial discretization resolution on prediction performance (Shanghai dataset).

As illustrated in Figure 9, the Top-1 Accuracy exhibits a clear upward trend as the grid size increases. This is expected, as a coarser grid results in fewer classes, reducing the perplexity of the classification task. Specifically, Acc@1 improves from 0.596 at 500 m to 0.749 at 2 km. However, this accuracy gain comes at the cost of spatial granularity—predicting a 2 km region provides less actionable guidance for navigation systems than a 500 m region. Interestingly, the regression performance remains relatively stable, achieving the lowest error at the Standard resolution (1 km). This suggests that the 1km scale optimally captures the spatial context correlations (e.g., “parking in a residential zone usually lasts overnight”) required for duration estimation. Consequently, we adopt the 1km resolution as the default setting to balance prediction accuracy with service utility.

### 5.7. Discussion

The experimental results above verify Spark’s superior performance in modeling long-term mobility patterns. To better position our work, we compare the proposed event-driven paradigm with classical Model Predictive Control (MPC) [26], a gold-standard method for vehicle trajectory tracking and dynamic planning [27]. Although MPC performs well in handling kinematic constraints and short-term trajectory execution based on physical laws, both experimental results and theoretical analysis indicate that the Spark architecture is more suitable for parking intent prediction, for three main reasons.

- MPC excels in physical control but cannot model subjective human intent, whereas Spark learns implicit decision rules from data. MPC uses explicit differential equations (e.g., kinematic bicycle models) to optimize future states, which works well for physics-based control but struggles with high-entropy human behaviors driven by subjective preferences. As a data-driven approximator, Spark captures these implicit rules directly from historical data without predefined cost functions.
- Spark offers stronger long-range temporal scalability than fixed-horizon MPC. MPC uses a fixed prediction horizon (e.g., 100 ms steps), which leads to excessive computational cost when predicting long parking durations from minutes to days. In contrast, Spark’s event-driven TA-LIF neurons decouple memory from simulation steps, efficiently modeling long time intervals without extra overhead.
- Spark is more suitable for edge computing than MPC due to low latency and linear complexity. MPC relies on iterative solvers (e.g., quadratic programming) at each step, creating high computational load for vehicular edge devices. Spark uses sparse spike-based accumulation with linear complexity, achieving real-time inference (46.2 ms) for resource-constrained smart cockpit systems.

In summary, while MPC remains indispensable for the low-level execution of vehicle motion, Spark provides the necessary high-level cognitive prediction that serves as the reference input for downstream control systems.

## 6. Conclusions

In this paper, we proposed an event-driven spiking neural network framework, called Spark, to address the problem of vehicle parking prediction. Extensive experiments on real-world datasets validate the effectiveness of our proposed model. First, the TA-LIF neuron with logarithmic discretization successfully balances temporal resolution with parameter learnability. Second, the accumulate-based readout strategy enables high-precision continuous value prediction while retaining the efficiency of neuromorphic computing. Finally, the SCG mechanism incorporates environmental factors via a lightweight gating strategy to enhance model robustness without disrupting the sparse computing paradigm.

In future work, we plan to validate the energy efficiency of Spark by migrating from simulation to physical neuromorphic hardware and to explore online learning mechanisms for personalized mobility modeling.

## Figures and Tables

**Table 1:** Statistical summary of the real-world vehicle trajectory datasets.

City	Time Period	Vehicles	Raw GPS Points	Parking Events	Location Classes
Shanghai	January 2023–January 2024	5246	332,568,498	3,824,512	4820
Shenzhen	January 2023–January 2024	7831	418,316,988	5,712,390	3950
Changsha	January 2023–January 2024	5071	365,469,915	3,698,045	2765

**Table 2:** Overall performance comparison (Mean ± Std). Best results are in **bold**, and second best are underlined. The results are averaged over 5 runs with different random seeds.

Dataset	Metric	Markov	DeepRP	Time-LSTM	ODE-RNN	THP	iTransformer	TCJA-SNN	Spark (Ours)
Shanghai	Acc@1	0.485±0.000	0.631±0.002	0.642±0.003	0.658±0.004	0.661±0.003	0.665±0.002	0.628±0.005	0.663± 0.002
	Acc@5	0.650±0.000	0.812±0.003	0.835±0.004	0.858±0.005	0.860±0.004	0.862±0.003	0.832±0.006	0.861± 0.003
	RMSE	1.680±0.000	1.250±0.010	1.160±0.020	1.070±0.030	1.060±0.020	1.020±0.010	1.290±0.040	1.050± 0.010
	MAE	1.120±0.000	0.850±0.010	0.780±0.010	0.720±0.020	0.700±0.010	0.690±0.010	0.880±0.030	0.700± 0.010
Shenzhen	Acc@1	0.492±0.000	0.631±0.003	0.645±0.003	0.660±0.005	0.665±0.003	0.670±0.002	0.632±0.006	0.668± 0.002
	Acc@5	0.665±0.000	0.825±0.003	0.842±0.004	0.865±0.006	0.869±0.004	0.875±0.003	0.840±0.007	0.870± 0.003
	RMSE	1.720±0.000	1.310±0.020	1.210±0.020	1.090±0.030	1.070±0.020	1.050±0.010	1.350±0.050	1.060± 0.010
	MAE	1.150±0.000	0.890±0.010	0.810±0.010	0.750±0.020	0.730±0.010	0.720±0.010	0.920±0.030	0.720± 0.010
Changsha	Acc@1	0.478±0.000	0.618±0.003	0.635±0.003	0.648±0.004	0.654±0.003	0.658±0.002	0.615±0.005	0.656± 0.002
	Acc@5	0.642±0.000	0.801±0.004	0.828±0.004	0.850±0.005	0.856±0.004	0.860±0.003	0.822±0.006	0.858± 0.003
	RMSE	1.650±0.000	1.280±0.020	1.180±0.020	1.080±0.020	1.050±0.020	1.030±0.010	1.330±0.040	1.040± 0.010
	MAE	1.090±0.000	0.860±0.010	0.790±0.010	0.750±0.020	0.730±0.010	0.710±0.010	0.890±0.030	0.720± 0.010

**Table 3:** Comprehensive efficiency and capacity comparison on Shanghai dataset. Energy is estimated based on 4.6 pJ/FLOP and 0.9 pJ/SOP [24]. Best results are in **bold**.

Model	Category	Params (M)	Inference Cost		Memory (MB)	Density (%)
			Ops (M)	Energy (μJ)		
DeepRP	RNN	**1.25**	2.40	11.04	**14.5**	100.0
Time-LSTM	RNN	1.38	2.85	13.11	16.2	100.0
ODE-RNN	Continuous	1.42	52.00	239.20	32.4	100.0
iTransformer	Transformer	3.42	14.50	66.70	48.6	100.0
TCJA-SNN	SNN	1.32	0.45	0.41	18.2	6.2
iSpikformer	SNN	3.15	0.92	0.83	42.0	5.5
**Spark (Ours)**	SNN	1.28	**0.38**	**0.34**	16.8	**4.8**

**Table 4:** Ablation study of key components in Spark. “×”: disabled, “✓”: enabled. When the Acc. Readout strategy is disabled (×), the model employs standard rate coding (spike count, scaled to the output range) for the final output representation. Results are shown as Mean ± Std, best results are in **bold**.

TA-LIF	SCG	Acc. Readout	Acc@1	RMSE
×	×	×	0.582±0.006 (+0.0%)	1.65±0.05 (−0.0%)
×	×	✓	0.608±0.003 (+4.5%)	1.35±0.02 (−18.2%)
✓	×	×	0.628±0.005 (+7.9%)	1.42±0.04 (−13.9%)
✓	✓	×	0.645±0.004 (+10.8%)	1.26±0.03 (−23.6%)
✓	✓	✓	0.663±0.002 (+13.9%)	1.05±0.01 (−36.4%)

**Table 5:** Ablation study of readout strategies. A decay factor of α<1.0 implies Leaky Readout, while α=1.0 represents the proposed ABR (Uniform Accumulation).

Readout Strategy	Decay α	Update Rule	Acc@1	RMSE
Leaky Readout (Strong)	0.5	$U_{t}=0.5U_{t-1}+s_{t}$	0.635	1.22
Leaky Readout (Mild)	0.9	$U_{t}=0.9U_{t-1}+s_{t}$	0.652	1.11
ABR (Ours)	1.0	$U_{t}=U_{t-1}+s_{t}$	0.663	1.05

**Table 6:** Fairness analysis of readout strategy (RMSE on Shanghai dataset). The “Standard” column cites the main results from Table 2 (using rate coding), while “With ABR” represents the baselines enhanced with our proposed readout.

Model	Readout Strategy (RMSE)		Spark (Ours)
	Standard	With ABR (Fair)	
TCJA-SNN	1.29	1.18	1.05
TS-LIF	1.15	1.10	

## Data Availability

The vehicle trajectory dataset is openly available on GitHub (https://github.com/HunanUniversityZhuXiao/PrivateCarTrajectoryData), accessed on 15 October 2025. Further inquiries can be directed to the corresponding authors.
